# Challenges and solutions for the downstream purification of therapeutic proteins

**DOI:** 10.1093/abt/tbad028

**Published:** 2023-11-19

**Authors:** Shuo Tang, Jiaoli Tao, Ying Li

**Affiliations:** GenScript ProBio Biotechnology Co., Ltd, Nanjing, Jiangsu 21100, P.R. China; GenScript ProBio Biotechnology Co., Ltd, Nanjing, Jiangsu 21100, P.R. China; GenScript ProBio Biotechnology Co., Ltd, Nanjing, Jiangsu 21100, P.R. China

**Keywords:** protein purification, fusion protein, recombinant protein, bispecific antibody, process development

## Abstract

The innovation in recombinant protein technology has brought forth a host of challenges related to the purification of these therapeutic proteins. This article delves into the intricate landscape of developing purification processes for artificially designed therapeutic proteins. The key hurdles include controlling protein reduction, protein capture, ensuring stability, eliminating aggregates, removing host cell proteins and optimizing protein recovery. In this review, we outline the purification strategies in order to obtain products of high purity, highlighting the corresponding solutions to circumvent the unique challenges presented by recombinant therapeutic proteins, and exemplify the practical applications by case studies. Finally, a perspective towards future purification process development is provided.

## Teaser

Statement of Significance: The diversity of protein structures has led to an increased complexity of product- and process-related impurities. The article summarizes the primary challenges encountered during the downstream processing of proteins, and presents effective solutions and case studies to tackle each major challenge.

## INTRODUCTION

Therapeutic proteins played a pivotal role in combating numerous diseases over the recent decades. Subsequently, the landscape of recombinant products has expanded exponentially, encompassing a diverse array of biologics, including blood products, hormones, cytokines and monoclonal antibodies (mAbs) [1]. With the advancement of genetic engineering technology, research in protein drugs achieved significant breakthroughs. The application of technologies such as fusion proteins, peptides and engineered antibodies diversified the construction of protein drugs, allowing for customization based on disease characteristics and patient needs [2]. Progress in protein expression systems has enabled the synthesis of virtually any desired protein or peptide [3]. Notably, this technological advancement has empowered the creation of unprecedented molecules that diverge from natural proteins, exemplified by fusion proteins and bispecific antibodies. These novel categories of therapeutic proteins exhibit unique functionalities that hold immense potential in enhancing drug efficacy.

In protein cell culture, there are impurities that can potentially trigger adverse reactions or allergic responses. Immune responses to these impurities can result in the loss of tolerance to their endogenous counterparts leading to serious adverse events [4]. The ICH Q6B guideline sets out the principles for consideration in setting product quality specifications (appearance, identity, purity and impurities, potency and quantity). Simultaneously, while navigating market price regulations and competition, pharmaceutical companies must address the vital challenges of cost-effective production and achieving high product recovery rate to ensure sustainable profitability.

Extracting high-purity target products from complex mixtures is an extremely challenging task. The diversity of protein structures has led to an increased complexity of product- and process-related impurities. Relying solely on lessons derived from mAbs has proven somewhat inadequate for the purification of complex recombinant proteins. For example, bispecific antibodies (BsAbs) are an emerging class of biotherapeutics with design diversity, each with a unique combination of antigen-binding domains that bind two different antigenic epitopes [5]. The multi-target design allows the simultaneous introduction of BsAb-specific byproducts, such as mispaired products, undesired fragments and higher levels of aggregates. The presence of these impurities that closely resemble the target antibody makes separation difficult, so it is usually necessary to develop additional purification strategies to obtain high-purity products. Antibody fusion proteins combine the advantages of antibody targeting and long half-life [6]. However, they suffer from chain mismatches, product cleavage and aggregates. Acidic instability can give rise to protein fragmentation or aggregation during affinity elution, thereby compromising protein quality. Acidic proteins with isoelectric points similar to host cell proteins (HCPs) make the separation of HCPs difficult. Recombinant proteins designed without tags lack specific affinity domains, thereby presenting significant obstacles in the removal of HCPs and the capture of the target product. In the face of these challenges, optimizing individual unit operations and the overall purification process becomes particularly important.

This review outlines the existing challenges encountered in the purification of intricate therapeutic proteins, including multispecific antibodies, fusion proteins and recombinant proteins. The main challenges in protein purification processes include protein reduction, protein capture, maintaining protein stability, removal of aggregates and HCPs and achieving high recovery [7–9]. The corresponding solutions that have been proposed to circumvent the unique challenges are presented. These approaches hold the potential to expedite the advancement of novel products in research and development, shorten the time required for commercialization and reduce the production cost significantly.

## SOLUTIONS FOR CHALLENGING PROTEIN PURIFICATION

Protein purification is a complex process involving multiple steps and diverse principles. The first unit operation is the removal of cells and cell debris from the culture broth. This essential step is referred to as “clarification” and can be achieved through physical methods, such as filtration. The purification process typically involves three primary steps: capture (isolation, concentration, volume reduction), intermediate purification (removal of bulk impurities or main protein contaminants) and polishing (removal of trace impurities, closely related contaminants and protein aggregates). These purification stages commonly utilize various chromatography techniques due to their excellent resolving capabilities. Finally, the product formulation involves obtaining the drug product through buffer exchange, and additives are added to enhance the stability and shelf life of the protein product.

This section summarizes the primary challenges encountered during the downstream processing of proteins, and presents effective solutions and case studies to tackle each major challenge.

### Protein reduction

Proteins contain multiple disulfide bonds, and the correct pairing of disulfide bonds is crucial for protein structure and activity. Ideally, disulfide bonds are properly paired before protein secretion to the extracellular environment, but in the complex oxidative-reductive environment of cell culture, disulfide bond breakage will occur easily [10]. In living organisms, the oxidation and reduction of disulfide bonds formed through the exchange of thiols and disulfides are catalyzed by thioredoxin (Trx) [11]. Release of sufficient intracellular enzymes, proteins and cofactors into the cell culture medium due to cell lysis can cause disulfide bond reduction [12]. The development of high-expression cell culture processes has resulted in an increasing occurrence of reduction of protein inter-chain disulfide bonds due to higher reductase release. Several studies have been conducted to understand disulfide bond reduction, yielding a range of recommendations to prevent its occurrence during the manufacturing process [10, 13].

**Figure 1:**
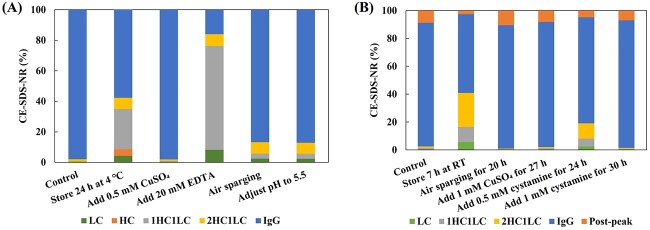
CE-SDS-NR results of samples under different conditions. (A) mAb molecule (pI = 7.2, Mw = 144 kDa). (B) BsAb molecule (pI = 7.8, Mw = 175 kDa).

Inhibiting the activity of reductase is an effective approach to address the antibody reduction issue. Our own practice demonstrated different resolution strategies for protein reduction (Fig. 1). Figure 1(A) illustrates a mAb molecule with severe reduction issues. During the clarification stage, it was observed that the antibody experienced significant molecular fragmentation after being stored at low temperatures for up to 24 hours, resulting in a decrease in the target molecule’s CE-SDS-NR purity from 97.2 to 57.7%. The reduction of antibody was effectively suppressed by adding 0.5 mM CuSO_4_ to the harvested samples. Copper ions can directly inhibit the activity of Trx, thereby suppressing enzyme-mediated molecular reduction [14]. Another BsAb molecule exhibits the same reduction issue, which becomes more severe with increasing incubation time (Fig. 1B). After 7 hours of incubation at room temperature, the CE-SDS-NR purity of the target antibody decreased from the initial 85.4 to 56.7%. Different strategies were employed to inhibit the reduction reaction, and the results demonstrated that continuous ventilation, addition of copper sulfate and cysteamine effectively suppressed molecular breakage. Air sparging is the preferred method due to its convenience in operation and absence of additives. Continuous air sparging can increase the dissolved oxygen in the liquid, reduce the generation of NADPH and maintain the electron source of thioredoxin in a deficient state, thereby inhibiting antibody redox reactions [15, 16].

In addition, inhibiting or slowing down antibody reduction can also be achieved by adjusting pH and temperature to reduce thioredoxin catalytic reactivity, as well as implementing rapid purification to reduce catalytic reaction time [10, 17]. Based on the mechanism of this system, Table 1 summarizes common strategies to address antibody reduction issue in downstream purification.

**Table 1:** Solutions for antibody reduction in downstream purification

Mechanism	Solution strategies
Inhibit Trx and hexokinase activity	1. Induce EDTA to inhibit hexokinase activity during cell harvest 2. Induce Cu^2+^, Zn^2+^ to inhibit Trx activity during cell harvest
Reduce reductant and enzyme amount	1. Induce oxidants such as O_2_, H_2_O_2_ and cysteine as competitors 2. Control depth filtration pressure to prevent enzyme release
Slow down enzyme reaction rate	Reduce pH and temperature before protein capture
Shorten enzyme reaction time	Reduce sample storage time before protein capture

### Protein capture

Protein capture is a recurring topic, and researchers have designed different tags on proteins to achieve effective separation [18]. While the capture of mAbs has been well-established, the capture methods for complex fusion proteins, tag-free recombinant proteins, and vaccines can vary significantly, depending on the unique properties of each protein.

#### Antibody fusion proteins

Affinity chromatography (AC) is widely used in the capture of mAbs, which relies on the specific binding between the target antibody with AC resin. As the complexity of molecular design increases, various antibody fragment-based fusion proteins have been developed, such as Fc-fusion proteins, ScFv-fusion proteins and VHH-fusion proteins.

**Figure 2:**
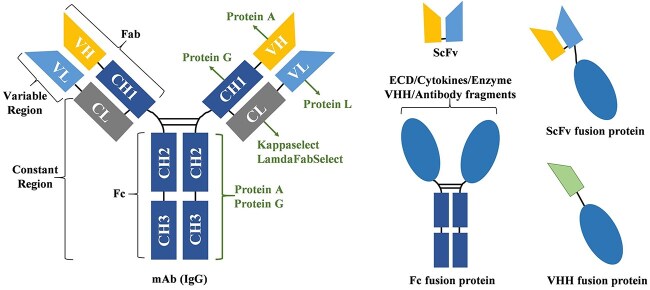
Schematic representation of IgG and fusion proteins classified based on their affinity binding domain.

As shown in Figure 2, antibodies are composed of different structural domains, and different domains can be designed and produced as recombinant fusion proteins with therapeutic activity. In antibody process development, Protein A, Protein G and Protein L AC have been developed for the capture of antibodies at different sites [7]. Fc-fusion proteins are the most common design structure and can typically be captured using Protein A and Protein G chromatography. Protein A capture is made possible through the interaction between Protein A and the Fc region of the target molecule as well as the heavy chain variable domain (VH) region of the HC for targets belonging to the VH3 gene family [19]. Protein G recognizes the Fab and Fc antibody regions in a manner similar to Protein A but with different binding specificities. The protein designed based on ScFv is another form of composition, and these forms are achieved through different combinations of chains and linkers. Protein L and Protein A can specifically recognize the VL and VH regions of ScFv for selective capture.

Selection of the optimal affinity resin is the primary task of protein capture. Theoretically, Protein A, Protein G and Protein L can all be used for capture of Fab fragment, they showed vastly different binding capability in applications. Different ligand structures, ligand densities and resin matrix led to significant difference in protein capture capability among same ligand types. While the principle can offer guidance for the selection of capture resins during process development, it is important to note that various resins may demonstrate distinct specificities for different molecules. Our own practice showed that Protein L resin demonstrated a superior capture ability for a Fab fragment, and Protein L resin from different sources displayed distinct capture capabilities. (Supplementary Fig. S1).

**Figure 3:**
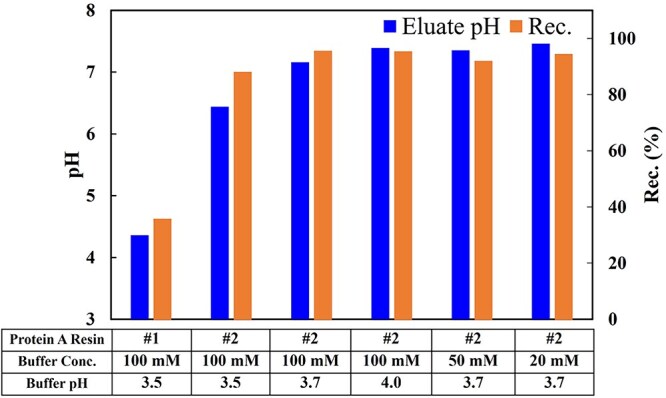
Effect of affinity resin and elution conditions on the eluate pH and recovery of acidic fusion protein (pI = 5.4, Mw = 111 kDa).

Besides the selection of resin, the choice of buffer system is also crucial for protein capture, especially for unstable fusion proteins. AC typically employs acidic elution to separate the target protein from the resin, under which condition the protein may become unstable. In addition, for acidic fusion proteins, the adjustment of pH across its isoelectric point (pI) may lead to protein precipitation or aggregation. Thus, for fusion proteins that are sensitive to acidic conditions, it is recommended to select a resin with lower affinity and reduce the buffering capacity of the elution buffer to increase the elution pH. Our own practice showed that the effect of affinity resin, elution buffer concentration and pH on the capture of an acidic fusion protein (Fig. 3). This protein precipitates when exposed to acidic conditions. Affinity resin#2 has been specifically designed to enable the elution of protein at high pH and avoid protein precipitation under acidic conditions. Additionally, the affinity resin#2 exhibits lower affinity to protein, allowing for high recovery during high pH elution. Reducing the buffering capacity of the elution buffer can further increase the pH of the protein eluate. After optimization, the pH of protein eluate remains above its pI throughout the entire capture process, thus avoiding post elution pH adjustment and product loss through aggregation.

#### Tag free recombinant protein

**Figure 4:**
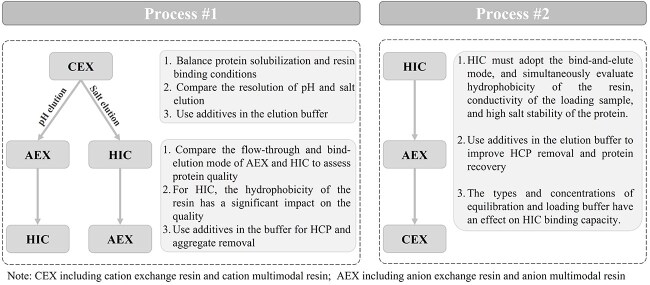
The purification process of tag-free recombinant protein.

Due to the lack of affinity domains, tag-free recombinant proteins can only rely on non-specific capture, typically based on the protein’s physicochemical properties such as charge, hydrophobicity and molecular weight. Figure 4 recommends the purification strategy for tag-free fusion proteins. Ion exchange chromatography (IEX) typically provides high resolution under mild conditions with high binding capacity, and is, therefore, recommended for capturing recombinant proteins. Excessive adsorption of pigments on anion exchange chromatography (AEX) resin causes a decline in product purity and makes it challenging to regenerate the resin, consequently reducing its operational lifespan. Hence, cation exchange chromatography (CEX) is used as the preferred ion exchange capture method. Additionally, the sample pH should differ by at least 0.5 unit from the isoelectric point of the target molecule to ensure product stability. The pH and conductivity need to be fine-tuned to maximize the binding capacity and separation. Hydrophobic interaction chromatography (HIC) resins have been employed in protein purification steps and offer major advantages such as high adsorption capacity, high selectivity, mild elution conditions, and low cost [20]. By selecting a resin with suitable hydrophobic strength and optimizing the high-salt loading condition, better separation effects can be achieved. Furthermore, HIC elution is performed under low-salt conditions, making it relatively friendly for subsequent processes.

**Figure 5:**
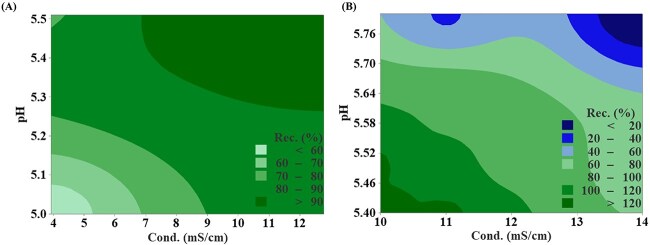
Counter plot of solution conductivity and pH versus recovery. (A) Recovery from protein precipitation at different pH and conductivity. (B) The CEX recovery at different loading pH and conductivity.

Our own practice demonstrated that the capture strategy of an acidic recombinant protein with a pI of 5.9 (Fig. 5). The protein solubility was influenced by pH and conductivity, with precipitation occurring at low pH and low conductivity. On the other hand, to enable capture by CEX, pH should be kept below the protein’s pI and conductivity should be low to maintain sufficient electrostatic interaction. Therefore, the loading condition needs to be carefully adjusted to enable protein capture while maintaining protein stability. From Figure 5(B), pH had a greater impact on recovery than conductivity within the test range. Based on these results, the loading conditions were set at pH 5.5 and conductivity of 11 mS/cm to achieve optimal capture capability.

### Unstable protein

Ensuring protein stability throughout the process is the primary consideration of protein purification, as most proteins are susceptible to changes in temperature, pH, or salt concentration [11]. Due to the complexity of artificially designed non-natural proteins, they are prone to issues such as precipitation and reduced quality during conventional purification process. The following summarizes the factors causing protein instability and the strategies to address this problem during protein purification process.

#### Buffer system and conditions

Inappropriate pH condition is the most common factor leading to protein instability, which results in rapid protein aggregation and fragmentation [21]. The elution buffer for AC is typically acidic, and the prolonged exposure to this condition leads to a sustained decline in protein activity. Therefore, upon elution, the pH should be adjusted back to neutral as soon as possible to prevent long-term activity reduction. In addition, the buffer system and protein concentration are also crucial for stability. For loosely folded proteins, inappropriate solution conditions can lead to partial exposure of hydrophobic regions, thereby accelerating the formation of aggregates.

#### Stabilizers

During the virus inactivation process, organic solvents have been employed as a solution to address the instability issue caused by low pH. However, the addition of inappropriate organic reagents can induce higher degree of aggregation formed through non-covalent binding. Addition of PS80 alone with proper concentration and treatment time can achieve the desired viral inactivation [22]. Surfactants not only have the ability to inactivate viruses but also helps to maintain protein stability. During the process development, significant precipitation occurred during the affinity step for proteins with strong hydrophobicity. Considering the relatively extended molecular structure and the presence of exposed hydrophobic regions in the molecule, a surfactant was introduced in the process buffer to stabilize the protein structure and enhance the product recovery significantly.

#### Other factors

Heat and shear forces can cause irreversible protein aggregation, while freezing and dehydration may result in conformational changes in some intricate protein structure and affect their activities. Although excipients are typically added to stabilize the drug substance (DS) stability during freeze-thaw, adding extra excipients in the process intermediate is not desirable during the purification process. Therefore, shortening the purification process duration and minimizing the sample freeze–thaw is the most effective and convenient approach.

Table 2 summarizes the factors causing protein structural changes during the purification process and their corresponding resolution strategies.

**Table 2:** The factors affecting protein stability during the purification process and the corresponding resolution strategies

Influence factors	Stage	Strategies
Molecular structure	All	Add stabilizers such as PS80 For proteins that are sensitive to metal ions, ensure the purity of the reagents and optimize the amount added
pH	AC	Shorten the storage time in low pH condition Choose high pH elution affinity resin or optimize elution buffer system
	VIN	Use S/D inactivation or other inactivation reagents
Ionic conditions	HIC	Screen highly hydrophobic resin to reduce the conductivity required during sample loading Try flow-through mode to avoid high conductivity loading
	All	Maintain optimal salt concentration for salt solubilized proteins
Buffer system	All	Choose the optimal buffer system
Freezing	Intermediates	Shorten the processing time to avoid freezing Add cryoprotectants without affecting the process
	DS	Add cryoprotectants
Temperature	All	Avoid high-temperature conditions, thaw at low temperature
Shear force	UFDF	Use low shear equipment, reduce operation pressure and add protectants

### Aggregate removal

Protein aggregation is one of the most challenging aspects in protein purification. Part of the aggregates originate from the protein formation during cell culture processes due to covalent or non-covalent interaction. The late-stage aggregation is largely dependent on buffer conditions, including pH, ionic strength, buffer system and additives [23, 24]. Different modes of chromatography are utilized to remove aggregates, depending on the variations in the physical and chemical properties between the target protein and the aggregates.

#### Affinity-based purification

**Figure 6:**
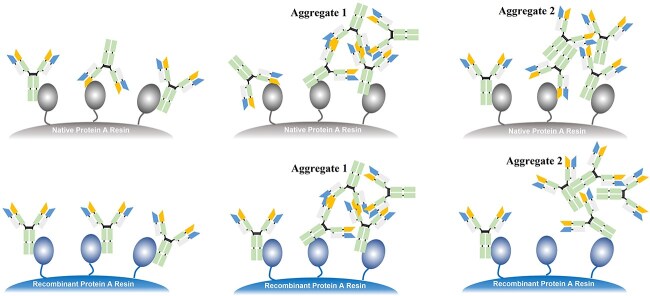
Protein A-mAb interaction. The polymer can simultaneously bind multiple ligands of the resin, while the mAb can only bind a single ligand. The binding site and binding capacity of aggregate and affinity resin depend on protein A and aggregate types.

Both monomers and aggregates can be captured by affinity resins. The differences in affinity can yield distinct separation effects on aggregates, as Protein A can simultaneously bind to the Fc and Fab regions of the antibody, and the binding affinity depends on the design of the resin and antibody [25]. As shown in Figure 6, the affinity between Protein A and antibody depends on the structure of both. Native-Protein A has weak interaction with the Fab region as well as strong binding with the Fc region. In contrast, recombinant Protein A lacks interaction with Fab. Studies found that each monomer can bind to only one Protein A ligand, while each dimer can bind to two ligands [26]. Therefore, aggregates of one mAb are more strongly retained compared with the mAb monomer. On the other hand, during aggregate formation, the Fc regions might be buried inside or partially lost [25]. For this type of aggregates, the interaction with recombinant Protein A becomes weaker than the interaction between mAb monomer and protein A. Frequently, incorporating suitable wash steps before elution can effectively eliminate these aggregates at an early stage.

Additives are commonly used to separate aggregates and target proteins. The addition of calcium chloride/polyethylene glycol (PEG) or sodium chloride/PEG combinations to the wash and elution buffers can effectively enhance the separation of aggregates and target antibodies [27]. The Hofmeister series of salts can improve resin selectivity by adjusting the hydrophobic interactions between antibody species and Protein A ligands. The use of magnesium chloride and calcium chloride as elution additives has resulted in excellent separation between bispecific products and Fc-Fc homodimers [28].

#### Charge-based purification

Aggregation gives rise to protein surface coverage, leading to disparities in surface charge when compared to individual monomers. Although aggregates are usually more tightly bound to the CEX resin than monomeric proteins, the separation between the two is generally difficult, especially when the aggregates are dimers [29]. Besides screening different types of cationic resin, the separation of monomers and aggregates can be improved by adding excipients to the mobile phase [30]. The utilization of a pH-conductivity hybrid gradient elution method has demonstrated its efficacy in eliminating aggregates from samples, if a consistent, linear and reproducible pH profile is provided [31].

#### Hydrophobicity-based purification

HIC presents notable benefits in aggregate removal, owing to the increased surface hydrophobicity exhibited by aggregates in comparison to monomers [32]. The efficacy of separation through HIC can be influenced by protein hydrophobicity, salt type and concentration, and hydrophobicity of the resin. By reducing the salt concentration or using resins with stronger hydrophobicity, the target protein can flow through while the aggregates and other impurities are retained, thus avoiding protein instability caused by high conductivity [33]. Additives with different polarities can alter the hydrophobic interactions among proteins, impurities and the resin, thereby improving resolution and achieving better separation.

#### Multimodal-based purification

Some proteins and aggregates cannot be separated effectively by using chromatography with a single mechanism. Multimodal chromatography allows for separation based on charge, hydrophobicity, hydrogen bonding, and other factors in a single step, and therefore provides better selectivity. Ceramic hydroxyapatite (CHT™) is one type of multimodal resin with a dual exchange mechanism based on phosphate groups for cation exchange and calcium ion metal chelation. Experimental results have demonstrated the excellent efficacy of CHT™ resin in removing aggregates, DNA, endotoxins, and other impurities [34]. Therefore, in the development of BsAbs, fusion proteins, and recombinant proteins, CHT™ resin can be considered for the removal of challenging impurities.

The formation mechanism of aggregates is complex, which leads to heterogeneous structural characteristics. Therefore, the removal of aggregates requires thorough optimization efforts. Table 3 summarizes the methods and optimization strategies for removing protein aggregates during the purification process.

**Table 3:** Strategies for aggregate removal using different types of chromatography

Type of chromatography	Mode	Optimization strategy
AC	B-E	1. Affinity resin screening 2. Wash buffer selection 3. Elution buffer selection 4. Additive selection in the elution buffer, such as polyols, amino acids and high-valent metal salts. 5. Elution pH optimization
IEX	F-T	Increasing pH to remove aggregates based on weak binding mode.
	B-E	1. Investigation of sample pH and conductivity to modulate protein binding. 2. Elution methods selection, exploring the resolution differences between salt elution and pH elution. 3. Combination of elution pH and conductivity as optimization factors. 4. Additive selection in the elution buffer, such as polyols and amino acids.
HIC	B-E	1. Selection of resin, where strong hydrophobic resins can be used for lower conductivity loading, and weak hydrophobic resins for higher conductivity loading. 2. Additive selection in the elution buffer, such as polyols and amino acids
	F-T	1. Selection of resin depending on hydrophobicity. 2. Sample pH and conductivity optimization.

Note: B-E, bind-and-elute; F-T, flow-through.

### HCP removal

HCPs are heterogeneous mixtures of proteins secreted by living cells and intracellular proteins released upon cell death and lysis, with the majority of HCP impurities being acidic proteins. Over 6000 HCPs derived from CHO cells have been identified through proteomics and other methods [35]. In downstream processes, protein purification typically involves the use of orthogonal chromatographic separation methods to achieve HCP removal. Nevertheless, achieving higher titers of such proteins during cell cultivation proves difficult, which consequently leads to higher HCP contents for tag-free recombinant proteins (> 10 000 ppm) and BsAb or fusion proteins (a few thousand ppm) as compared with typical mAb (< 2000 ppm) after capture.

#### Depth filtration

Depth filtration (DF) is used for solid–liquid separation and can also be employed for HCP removal utilizing electrostatic interaction between the charged binder and proteins. Therefore, two separation strategies are employed: first, controlling the conditions to promote HCP precipitation; and second, removing soluble HCPs through electrostatic interaction with the depth filter material. The pI of the majority of CHO cell-derived HCPs falls within the pH range of 4.5–7.5, and they become less soluble during the neutralization process, leading to aggregation and precipitation. In mAb development, controlling the pH after low pH inactivation allows HCPs to precipitate from solution, enabling their removal during intermediate depth filtration [36]. An optimal process with pH adjustment can be designed to achieve selective precipitation of HCPs while minimizing product loss.

#### Affinity chromatography

AC is an ideal tool for protein capture, as the target protein can be strongly bound to the resin in a specific manner. However, some HCPs are co-eluted with the products due to non-specific interaction with the Protein A resin or non-specific binding with the target proteins [37]. A common approach is to use wash buffer with high salt concentration or low pH (5–5.5) to remove a part of the HCP prior to elution, but these conditions often do not completely disrupt the binding between HCPs and the target protein. Recent studies have found that using high pH and high conductivity wash buffer solutions can remove HCPs more effectively, as the electrostatic interactions between the mAb and most HCPs are repulsive under alkaline conditions (pH > 8) [36]. Since HCPs can bind to proteins through various mechanisms, including electrostatic interactions, hydrophobic interactions, and hydrogen bonding, reagents that disrupt these interactions can be used to effectively remove HCPs. To date, wash buffer solutions containing different additives have been tested for HCP removal [38–40]. Histidine hydrochloride has shown good HCP removal results, as it can form hydrogen bonds with proteins, disrupting hydrophobic and electrostatic interactions [40].

#### Ion exchange chromatography

**Figure 7:**
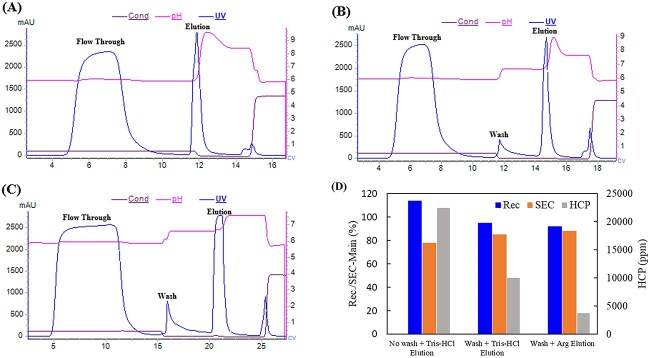
The CEX chromatograms and quality results for different wash and elution conditions for a tag-free recombinant protein. (A) No wash, elution buffer: Tris-HAc. (B) Wash + elution buffer: Tris-HAc. (C) Wash + elution buffer: arginine. (D) The results for different wash and elution conditions.

The separation of HCPs is attributed to charge–charge interactions. Most HCPs belong to acidic proteins, so AEX in flow-through mode is effective for HCP removal in the case of basic target proteins. In the flow-through mode, weak partitioning chromatography (WPC) has been proposed for the purification of mAbs using a relatively high pH to enhance HCP removal [41]. For the bind-and-elute mode, the pI differences between HCPs and target proteins can be exploited to remove parts of HCPs prior to protein elution, thus minimizing co-elution of HCPs with the target protein. Additionally, amino acids have been used in various chromatographic separations to enhance the clearance of HCP during mAb purification processes [42, 43]. Our own practice showed the use of CEX in combination with additional wash steps and amino acid additives in the elution buffer to achieve effective HCP removal for a recombinant protein with a pI of 5.9 (Fig. 7). The increase of wash buffer strength induces a partial loss of the target protein, but it proves effective in removing weakly bound HCPs. The addition of arginine in the elution buffer further reduces HCP content. The presence of arginine stabilizes the pH after protein elution, preventing the elution of more strongly bound HCPs. Therefore, by adding a wash step and optimizing the elution buffer, the HCP removal capability can be significantly enhanced.

#### Hydrophobic interaction chromatography

Amino acids, which are the building blocks of proteins, differ in both charge and polarity, resulting in variations in the hydrophobicity between the target protein and HCP. HIC in both bind-and-elute mode and low salt flow-through mode have demonstrated great HCP removal capabilities [44]. The selectivity can be improved by controlling the type and concentration of salt, temperature, pH and ligands used in the HIC process. Figure S2 summarizes the effects of HIC in HCP removal for our selected platform projects. Both the bind-and-elute mode and the flow-through mode can achieve good HCP removal, with an average removal rate of over 90%.

#### Multimodal chromatography

Multimodal resins possess dual properties of ionic exchange and hydrophobicity, the combination of multiple interactions may provide better selectivity. Capto™ Adhere resin, harnessing both anion exchange and hydrophobic interaction functionalities, could achieve a clearance capability of up to 2–3 LRV to reduce HCP levels below 10 ppm [45]. Capto™ MMC resin, with both cation exchange and hydrophobic properties, is typically used in bind-and-elute mode and has demonstrated good HCP and impurity removal capabilities under optimal process conditions [46]. CHT™ resin, operating in flow-through mode, has also exhibited excellent competence in HCP removal [47]. However, achieving enhanced separation through multimodal chromatography requires comprehensive Design of Experiments study to explore the potential interaction of different factors.

**Figure 8:**
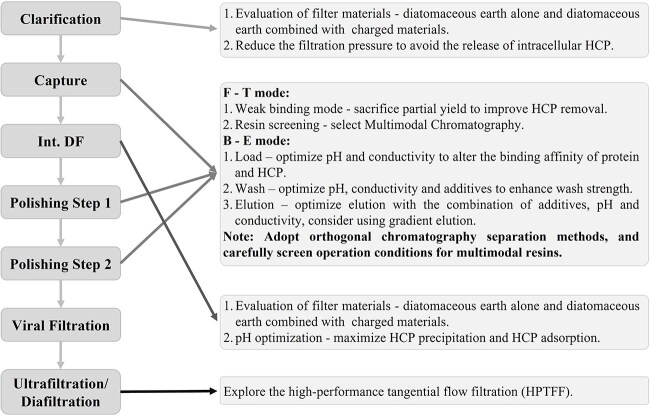
Effective strategies for HCP removal at different stages of downstream processing.

Among all impurities related to the process, HCPs are usually of particular concern because they can affect the safety, efficacy and stability of the product. Recently, the development of high density and perfusion cell culture has introduced more challenges in the purification of increased level of HCPs and other impurities. Figure 8 recommends effective strategies for HCP removal at different stages of downstream processing. The purification should be optimized towards maximizing the overall removal capability of the process.

### Protein recovery

For complex proteins, the presence of a significant level of aggregates and impurities such as HCPs leads to lower yields. Therefore, maximizing recovery while maintaining product quality becomes a pivotal challenge for protein purification process development.

DF is used for the initial clarification of cell culture broth to protect chromatography columns. Selecting appropriate DF filter is key to reduce the protein loss due to adsorption. Uneven charges on a portion of the protein surface result in electrostatic interactions with the charged membrane, causing protein adsorption. Hence, investigation of the DF membrane with different materials improves the protein recovery rate and reduces loss from protein adsorption. Besides, partial adsorption can be mitigated by increasing the conductivity of the wash buffer. Nevertheless, special attention should be given to the adjustment of wash buffer salt concentration, as excessively high conductance is potentially unsuitable for the subsequent separation phase.

In AC, strong binding of protein on the resin may make the elution of protein difficult, leading to low protein recovery. Previous studies have shown that the presence of high ionic strength may promote hydrophobic interactions between protein and Protein A, therefore requires lower pH for elution [48]. Additionally, fusion proteins generally exhibit weaker stability compared with mAbs, especially under low pH conditions, which pose challenges for elution at lower pH. By using arginine buffer or other additives, elution can be achieved at high pH while ensuring high recovery rate. HIC is often associated with low yield due to unfavorable solvent conditions, or denaturation of target protein on the hydrophobic surface. Various methods have been explored to improve the recovery rate of HIC. In HIC, bind-and-elute mode frequently led to overly strong protein binding, posing challenges in protein elution. Arginine and hexylene glycol can weaken the interactions between proteins and the resin matrix, thereby improving the recovery rate and purity of various proteins [49].

While striving for high purity by addressing issues such as the removal of aggregates and HCPs, part of the protein product is inevitably lost. Based on the data from our platform, protein loss primarily occurs during depth filtration and various chromatography steps. For depth filtration, selection of appropriate membranes and optimization of wash buffers are the main measures to address the protein loss. For chromatography, the addition of excipients in the product intermediate and process buffer is an effective strategy to improve the recovery by modulating the binding affinity between proteins and resins.

## FUTURE PERSPECTIVES AND CONCLUSION

The development of therapeutic protein drugs is a multifaceted systemic engineering endeavor that involves diverse disciplines. In comparison to the substantial advancements seen in upstream processing areas such as molecular design and cell line engineering, there have been relatively fewer reports on the development of protein downstream purification methods. As we have discussed in this review, the diversity of BsAbs, fusion proteins, and recombinant proteins presents a significant challenge in developing effective and economical purification processes.

Chromatography techniques play a pivotal role in protein purification; however, the development of resins lags behind the demand arising from antibody technology advancement. Affinity resins and multimodal resins represent the most promising chromatographic media for addressing challenges in protein purification. Both types of resins exhibit stronger selectivity for impurities, enabling better differentiation between different proteins and process-related impurities. In contrast, progress in the development of filtration steps has been slow-paced, making ideal impurity removal based on filtration processes difficult to achieve. Nevertheless, the combination of resins and filtration processes in product design may offer a promising direction for breakthroughs in consumables. Such products would merge the efficiency of filtration processes with the selectivity of chromatographic processes, potentially enhancing the efficiency and economy of purification significantly.

In addition to anticipating suppliers to provide improved purification consumables, efficient experimental design is another pursuit in the development of purification processes. The design of protein purification processes is heavily based on empirical knowledge and a large number of trial-and-error experiments, often resulting in suboptimal overall processes and low utilization of raw materials. Addressing this issue primarily involves reducing the number of experiments and increasing experimental efficiency. Rational experimental design is the basis for reducing the number of experiments, requiring sufficient understanding of the target protein. Computational biology represents a future trend that leverages computer models and machine-learning algorithms to predict the properties of target proteins, identify correlations and optimize purification process conditions, thereby reduces trial-and-error experiments. This has the potential to fundamentally enhance the accuracy and efficiency of purification process development. On the other hand, the introduction of high-throughput screening technology has greatly improved the efficiency of protein purification. High-throughput screening accelerates the evaluation, optimization and selection of purification processes by parallel processing a large number of samples. The combination of computer-aided design and high-throughput screening is an excellent option for future purification process development, which is worth looking forward to.

In summary, this overview demonstrates that challenges still exist in seeking to improve the development of bioprocess purification, while also highlights the significant progress that has been made. Through the development of rational processes, we can address the majority of challenges to meet regulatory requirements. With the continuous advancement of technology, the exploration of more efficient, accurate and environmentally friendly protein purification methods will continue to drive the development of this field.

## Supplementary Material

Supplementary_Data_tbad028

## Data Availability

The authors confirm that the data supporting the findings of this study are available within the article.
