# Supplementary material for: Challenges and solutions for the downstream purification of therapeutic proteins
Source: Antib Ther. 2023 Nov 19;7(1):1–12. doi: 10.1093/abt/tbad028 (PMC10791043; doi:10.1093/abt/tbad028)
Supplement: Supplementary_Data_tbad028 [file supplementary_data_tbad028.docx]

**Challenges and solutions for the downstream purification of therapeutic proteins**

**Shuo Tang, Jiaoli Tao, Ying Li***

GenScript ProBio Biotechnology Co., Ltd., Nanjing, Jiangsu, 21100, P.R. China

**Supporting Information**

Figure S1. Comparison of Fab fragment loading capacity of different resins. Protein L resin was the only one that demonstrated superior capture of a Fab fragments (pI 8.7, Mw 48 kDa). However, its capture ability was also influenced by the protein L structures, densities and resin matrix.

Figure S2. Removal of HCP using HIC for different proteins.


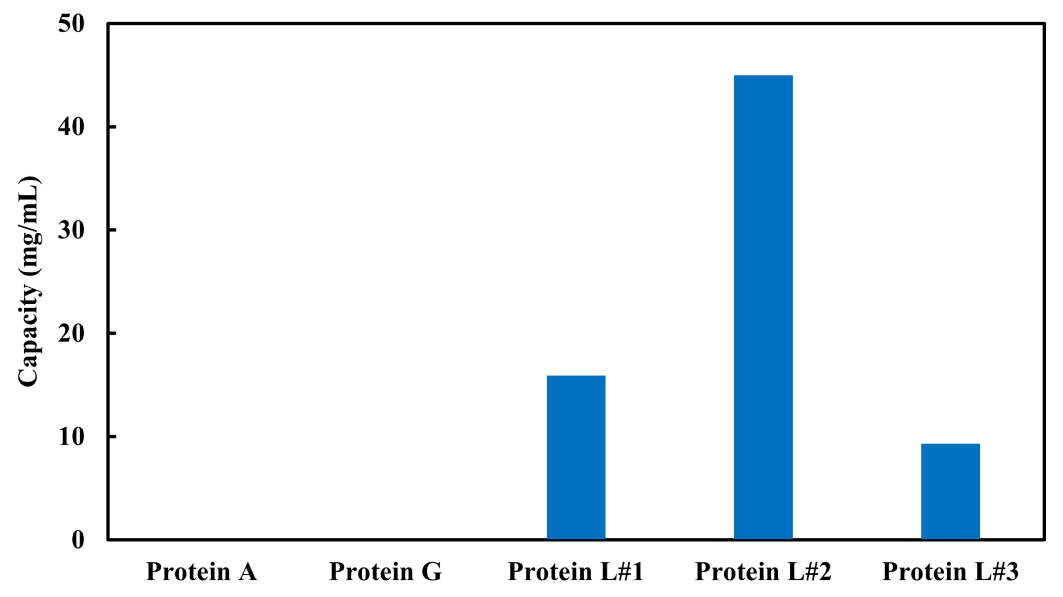


Figure S1. Comparison of Fab fragment loading capacity of different resins. Protein L resin was the only one that demonstrated superior capture of a Fab fragments (pI 8.7, Mw 48 kDa). However, its capture ability was also influenced by the protein L structures, densities and resin matrix.


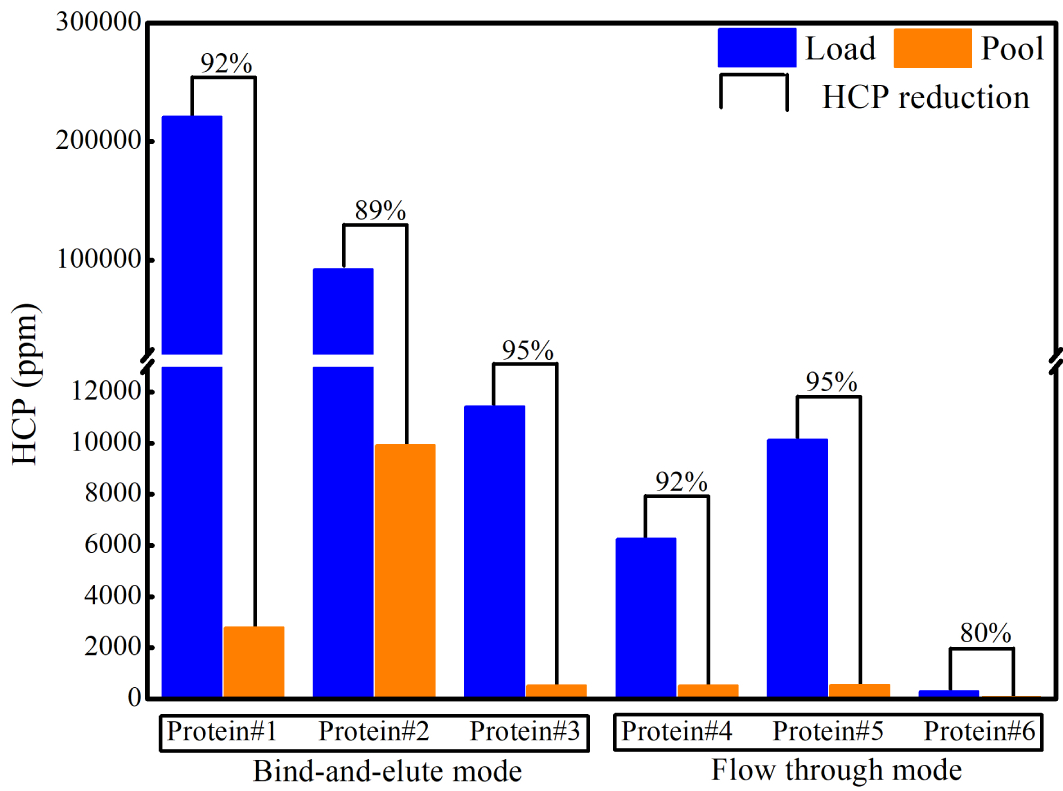


Figure S2. Removal of HCP using HIC for different proteins**.** Protein#1 (pI 6.4, Mw 107 kDa), Protein#2 (pI 6.4, Mw 216 kDa), Protein#3 (pI 5.8, Mw 71 kDa), Protein#4 (pI 8.6, Mw 170 kDa), Protein#5 (pI 5.7, Mw 66 kDa) and Protein#6 (pI 6.6, Mw 168 kDa)
